# Selected Spectroscopic Techniques for Surface Analysis of Dental Materials: A Narrative Review

**DOI:** 10.3390/ma14102624

**Published:** 2021-05-17

**Authors:** Katarzyna Kaczmarek, Andrzej Leniart, Barbara Lapinska, Slawomira Skrzypek, Monika Lukomska-Szymanska

**Affiliations:** 1 Department of Inorganic and Analytical Chemistry, Faculty of Chemistry, University of Lodz, 12 Tamka St., 91-403 Lodz, Poland; andrzej.leniart@chemia.uni.lodz.pl (A.L.); slawomira.skrzypek@chemia.uni.lodz.pl (S.S.); 2 Department of General Dentistry, Medical University of Lodz, 251 Pomorska St., 92-213 Lodz, Poland; barbara.lapinska@umed.lodz.pl

**Keywords:** dental materials, dental ceramics, spectroscopy, IR, FT-IR, Raman spectroscopy, UV-Vis, X-ray spectroscopy, XRF, XRD, MS

## Abstract

The presented work focuses on the application of spectroscopic methods, such as Infrared Spectroscopy (IR), Fourier Transform Infrared Spectroscopy (FT-IR), Raman spectroscopy, Ultraviolet and Visible Spectroscopy (UV-Vis), X-ray spectroscopy, and Mass Spectrometry (MS), which are widely employed in the investigation of the surface properties of dental materials. Examples of the research of materials used as tooth fillings, surface preparation in dental prosthetics, cavity preparation methods and fractographic studies of dental implants are also presented. The cited studies show that the above techniques can be valuable tools as they are expanding the research capabilities of materials used in dentistry.

## 1. Introduction

For centuries in dentistry, the search remains for the best methods and materials to restore missing tooth structures or replace missing teeth [1,2,3]. Modern dentistry, in addition to its primary role to improve the health of masticatory system, increasingly focuses on the aesthetics of the preformed reconstructions [4,5,6,7]. The rapid development of this field is possible thanks to the constant introduction of new materials and research techniques [8,9,10,11,12,13,14]. Due to wide range of (ceramic, metallic, synthetic or composite) dental materials available on the market, it is crucial that dental technicians and/or dentists choose the appropriate method by taking into account its limitations and features [13,15,16,17,18,19,20].

The application of the material in the oral cavity, i.e., in direct contact with tissues, places special demands on this group of biomaterials regarding their physicochemical and biological properties, i.e., biocompatibility, local and general harmlessness to the organism [18,21,22], resistance to the effects of physicochemical factors in the oral cavity [21] and biophysical indifference [18,19]. In addition, the accuracy in mimicking natural tooth shapes [19], stability of mechanical properties [19,22], ease processing [21,23], appropriate aesthetics [19,24], and finally a moderate price [19,23] are further requirements that need to be met. The first phenomenon that occurs after the introduction of the biomaterial into the oral cavity is the formation of a biofilm on its surface [25,26,27]. In order to prevent the micro-leakage and the biofilm formation, it is important to know the material structural and chemical components [7,28,29]. Surface structure, roughness, chemical composition and reactivity are just a few of the main characteristics that should be assessed before qualifying particular material for dental applications [18,19,27,30,31]. In addition, surface characteristics are related to other properties of the material, such as mechanical or chemical features, enabling detailed understanding how the material behaves under oral conditions [32,33].

The advanced development of microscopic and spectroscopic techniques enables a detail study of the chemical structure and surface phenomena [20,34] of studied materials. Microscopy enables observation of surfaces, and structural and intra-material changes at high magnification [35,36,37]. It includes, inter alia, Optical Microscopy [38,39,40], Electron Microscopy [39,40,41,42], X-ray Microscopy [39,40], and Scanning Probe Microscopy [39,41].

These techniques allow for advanced experimental research, which in combination with powerful acquisition procedures and data processing, enable detailed analysis of the studied system. Besides morphological and structural characterizations, microscopic techniques provide additionally information on the quantitative and qualitative chemical composition of the analysed sample either at a selected point, or along a given line, thus providing elemental maps (Energy Dispersive X-ray Spectroscopy—EDS), crystal orientation (Electron Backscatter Diffraction—EBSD), and many other characteristics of dental biomaterials [40,43,44,45]. The use of microscopic methods for material testing is widely described in the literature; therefore, in this paper we present the application of spectroscopic techniques, basic principles of operation, their advantages and limitations, as well as their application for studying dental materials.

Spectroscopy covers a lot of techniques based on the interaction of the electromagnetic radiation with the matter; sometimes, the term spectroscopy refers to an analytical technique involving generation and interpretation of spectra. The use of spectroscopy, especially in combination with microscopic techniques, provides valuable information on the chemical structure of dental materials. The major advantages of spectroscopic techniques are seen in the fact that they are non-destructive and using small amount of sample weights [34,46,47,48,49,50,51,52]. Spectroscopic techniques including: Infrared spectroscopy (IR), Fourier Transform Infrared Spectroscopy (FT-IR), Raman spectroscopy, Ultraviolet and Visible spectroscopy (UV-Vis), X-ray spectroscopy, and Mass Spectrometry (MS) are very useful in the dental material studies [53].

The aim of the current review was to provide the reader with a “broad view” of spectroscopic techniques and their application in a dental material research.

## 2. Search Strategy

An electronic search was carried out in SCOPUS and PubMed digital databases using the keywords related to the topic search and combining the keywords using “AND” and “OR”. The search strategy employed was as follows: (Dental material spectroscopic methods) OR Dental material spectroscopy) OR Dental ceramic spectroscopic method) OR Dental ceramics spectroscopy) OR Dental implant spectroscopy) AND (Dental material IR) OR Dental ceramic IR) AND (Dental material FTIR) OR Dental material FT-IR) OR Dental ceramic FTIR) AND ((Dental material Raman spectroscopy) OR Dental ceramic Raman spectroscopy) AND (Dental material UV-Vis spectroscopy) OR Dental material UV-Vis) Dental ceramic UV-Vis spectroscopy) AND (Dental material X-ray spectroscopy) OR Dental ceramic X-ray spectroscopy) AND (Dental material Mass Spectrometry) OR Dental ceramic Mass Spectrometry).

The review of potentially eligible data articles on a given spectroscopic technique was narrowed down to books, book chapters and overviews in Subject Area-Dentistry. If no information could be found in this regard, it was extended to research articles on chemical and material subject fields. The review is mainly based on articles on spectroscopic methods excluding microscopic methods.

Initially 12,904 articles were found. After removal of duplicates and restriction to the years 2008–2021, 1534 articles were identified from digital databases and a manual search. Full texts of papers were obtained from the journals. The inclusion and exclusion criteria for articles are presented in Table 1.

## 3. Fundamentals and Division of Spectroscopy

For the purpose of a comprehensive overview of spectroscopic techniques, it is necessary to apply various criteria for classification of the techniques. Most often, the following classification criteria are adopted: the range of electromagnetic radiation, properties of the studied systems, and the method of collecting a spectrum, referring to the way of energy exchange between the radiation and the matter. The division of spectroscopy according to the radiation range is actually related to various experimental techniques as listed in Table 2. Depending on the radiation range, different radiation sources, detectors and dispersion devices are used. In optical spectroscopy, prisms and diffraction gratings are most frequently used as dispersion devices [54,55,56,57].

Spectroscopy can be analysed based on the intrinsic aspects of the studied process. In this regard, the following types of spectroscopy can be differentiated: nuclear spectroscopy, atomic spectroscopy, and molecular spectroscopy with particular emphasis on the spectroscopy of condensed systems. Such division is related to the specific energy levels taking part in the energetic transition of the studied system. Each type of spectroscopy is associated with specific motion of the constituents of the system at a microscopic level, and thus differs in the magnitude of the energy between transition energetic states [34,53,58,59].

Depending on the radiation measurement process, one distinguishes three types of spectra: absorption, emission and Raman spectroscopy, respectively. Absorption spectra can be defined as a set of all transitions from lower to higher levels so, they correspond to an increase of the system energy (photon uptake). The simplest type of absorption spectrum arises when the lowest energy level, i.e., the basic level, is occupied. The occupation of energy levels is related to the thermodynamic equilibrium, which is determined by the temperature of the system. It is assumed that only the basic level is filled at room temperature. Emission spectra can be defined as a set of all transitions from higher to lower energy levels. Transitions in emission spectra correspond to the reduction of the energy, i.e., the radiation of photons. The characteristic feature of Raman spectra is the change in the frequency of the scattered radiation (νr) in relation to the frequency of the incident radiation (νp) [60,61].

## 4. Infrared Spectroscopy (IR) and Fourier Transform Infrared Spectroscopy (FT-IR)

### 4.1. Principle of the Technique

The area of the electromagnetic spectrum with the wavenumber (reciprocal of the wavelength) from approx. 14,000 to 200 cm^−1^, i.e., between the visible and the microwave region, is called the infrared (IR). The absorption of such energy amount within this region is large enough to cause the chemical bonds to oscillate, but not enough to cause their breakage. Molecules rotate around their symmetry axes and at the same time their atoms oscillate between equilibrium positions. The IR absorption spectra are obtained by measuring the relative intensity of the transmitted or absorbed radiation as a function of the wavenumber, and they are presented in form of a plot of the transmittance or absorbance of the radiation versus the wavenumber (cm^−1^).

There are many types of IR spectrophotometers, e.g., filter photometers, double-beam spectrophotometer, Fourier transform spectrometer, attenuated total reflectance (ATR) FT-IR instrument [55]. In modern Fourier transform devices, which consists of illuminating simultaneously the sample with a beam of radiation from the entire tested IR range. After this beam has passed through the sample, the beam from the same source has not been interfered with, and the spectrum is obtained using the Fourier transform of the recorded interference spectrum. This requires the use of equipment with a software that performs this mathematical operation and provides information about vibrations in the form of an interferogram. FT-IR spectroscopy allows to determine the characteristic vibrations of atomic groups of the studied compound.

Due to the fact that the FT-IR spectrum is characteristic for a given substance, infrared spectrophotometric analysis is most often used for qualitative analysis. In addition to characterizing a pure substance by means of FT-IR analysis, the presence of additional substances in a studied mixture, as well as interactions between atomic groups of different compounds in the mixture can be examined. Changes in the spectrum (presence of additional peaks) indicate the presence of other functional groups (presence of another compound), while the shifting of the peaks relative to the spectrum of the original pure compound indicates interactions of a given atomic group with other groups of the studied mixture [61,62]. Besides, the following infrared spectroscopic techniques can be currently differentiated: transmission spectroscopy (TS), internal reflection spectroscopy (IRS), which is also referred to as attenuated total reflection (ATR), mirror reflection (ERS—external reflection spectroscopy), diffuse reflection spectroscopy (DRS), emission spectroscopy (ES) and photoacoustic spectroscopy (PAS) [63,64].

### 4.2. Type of Tested Samples

In infrared spectroscopy substances can be examined in a gaseous, liquid and solid state. Gas-sample spectra can be obtained by inserting the gaseous sample into a purged cuvette. Liquids can be tested in a pure form, or in a solution form. Liquid samples are placed between the sodium chloride plates; approx. One to ten milligrams of a liquid substance is needed. For liquids that dissolve sodium chloride, silver chloride plates can be used instead. Solid samples are typically tested in a form of suspension, pellet, or deposited glassy film (a solid film on a glass substrate). The spectrum of a solid sample most frequently is obtained by dispersing the sample within an alkali halide pellet. The spectra of solids can also be obtained by preparing a film of the solid sample, following evaporation of the solvent from a droplet deposited on a sodium chloride plate of a solution containing the studied compound [55,65].

### 4.3. Sample Characteristics

FT-IR spectroscopy allows to determine the characteristic vibrations for groups present in a compound. Due to the fact that the FT-IR spectrum is a spectrum characteristic for a given substance, infrared spectrophotometric analysis is most often used for qualitative research. In addition to distinguishing between pure substances by means of FT-IR analysis, it is possible to investigate the presence of additional substances as well as their interactions with individual groups of the original compound. Changes in the spectrum (presence of additional peaks) indicate the presence of other functional groups (presence of another compound), while shifts in the directions of other wavelengths than for the spectrum of the original sample indicate the interaction of a given group with a different group of the added substance [61,62,66].

For example, the FT-IR method was used to identify the structure of a dental material, i.e., fibroin thanks to application of FT-IR, a NH functional group in fibroin was recognised as an amino acid structure with a peak of 3309.80 cm^−1^ [67]. Measurement of absorbance using FT-IR led to a spectra of the various bioceramic powders and finally their identification [68].

### 4.4. Advantages and Limitations

Currently, infrared spectroscopy is widely used technique to identify functional groups and chemical compounds (both organic and inorganic), as well as to assess the purity of a compound. It is inexpensive, instruments are easy to be operated, and IR spectra are obtained quickly [55]. Infrared spectroscopy is a promising alternative to other techniques, as it is not time consuming with respect to sample preparation, measurement and interpretation of the results; it is also non-invasive and relatively simple measuring technique. As all frequencies are measured simultaneously in FT-IR spectroscopy, the spectrum is obtained for a few seconds, being advantageous in comparison with conventional infrared spectroscopy measurements lasting [34,69,70,71]. The undoubted advantage of infrared spectroscopy techniques is the ability to test small amounts of the material [62], and in ATR technique, the ability to penetrate of the light beam an into a sample depth of about 0.5–3 µm [72].

The limitation of IR is the fact that in classic spectrometers, the IR spectra are obtained by examining the absorption of a specific beam of monochromatic radiation (one wavelength), and then sweeping the sample by gradually changing the wavelength during measurement with a dispersion element (prism, diffraction grating). Before the measurement, it is often necessary to properly prepare the sample, e.g., grinding the test material, thoroughly mixing with the potassium bromide (KBr) matrix and compressing under pressure to form a tablet. The sample should not contain water, because water strongly absorbs radiation with the wavenumber from approximately 3700 cm^−1^ and 1630 cm^−1^ (this absorption may obscure the bands of the tested substance) [55].

In the ATR sampling system, the main source of errors during the measurement is the imperfect contact between the sample and the diamond crystal [65]. The next drawback of FT-IR instruments is the fact that they are equipped only with a single beam, whereas dispersive instruments generally have a double beam [69].

### 4.5. Applications

IR spectroscopy was used to track the polymerization kinetics of dental resins [73,74] and adhesives [75,76,77,78] to improve the mechanical features of the dental material. The IR technique was also used to determine the role of intermolecular collagen cross-linking in the mechanical behaviour of dentin [79], to evaluate the structure of heterocyclic compounds as candidates for pulp regeneration [80] and to analyse new generation biomimetic materials replicating the mineral organic dentin and enamel complex [81].

The FT-IR technique was used for the structural characterization of dental materials such as: implant materials [82], biopolymers [83], ceramics [84], resin nanocomposites [85], implant coatings [86,87,88], bioceramics [89], resins [90,91,92,93,94], cements [95], bioglass [96], and self-curing materials [97].

### 4.6. Spectrum Example

**Figure 1:**
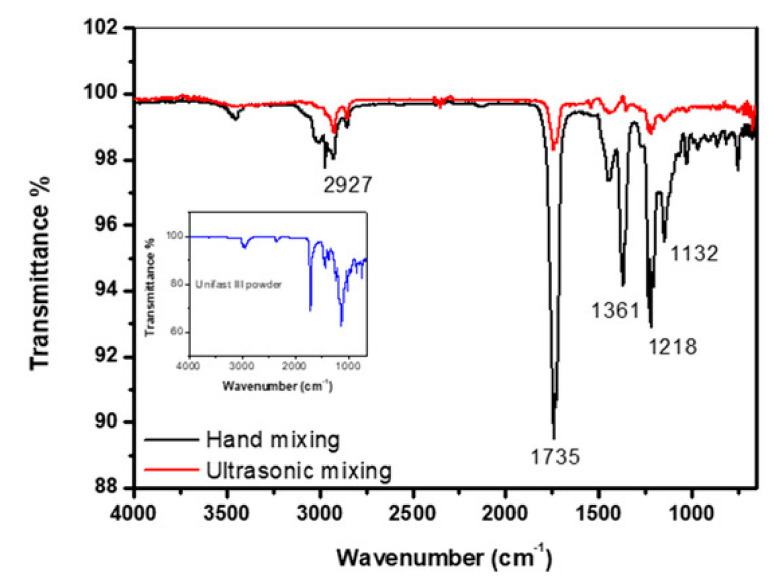
FT-IR spectrum of control specimen made by hand and ultrasonic mixing methods as well as the spectrum corresponding to UNIFAST III powder [97].

Figure 1 shows the FT-IR spectrum of self-curing polymethyl methacrylate (PMMA)-based dental materials (UNIFAST III, GC Corporation, Tokyo, Japan) control samples prepared by mixing UNIFAST III resin powder (GC Corporation, Tokyo, Japan) with UNIFAST liquid monomer (GC Corporation, Tokyo, Japan). The powder spectrum of GC UNIFAST III is depicted in the inset of Figure 1. The band at around 1132 cm^−1^ is the characteristic absorption vibration of PMMA. The bands at about 1218 cm^−1^, 1361cm^−1^, 1735 cm^−1^ and 2927 cm^−1^ are assigned to υ(C–O) stretching vibration, wagging vibration of C–H, C=O stretching, and C–H stretching, respectively [97].

**Figure 2:**
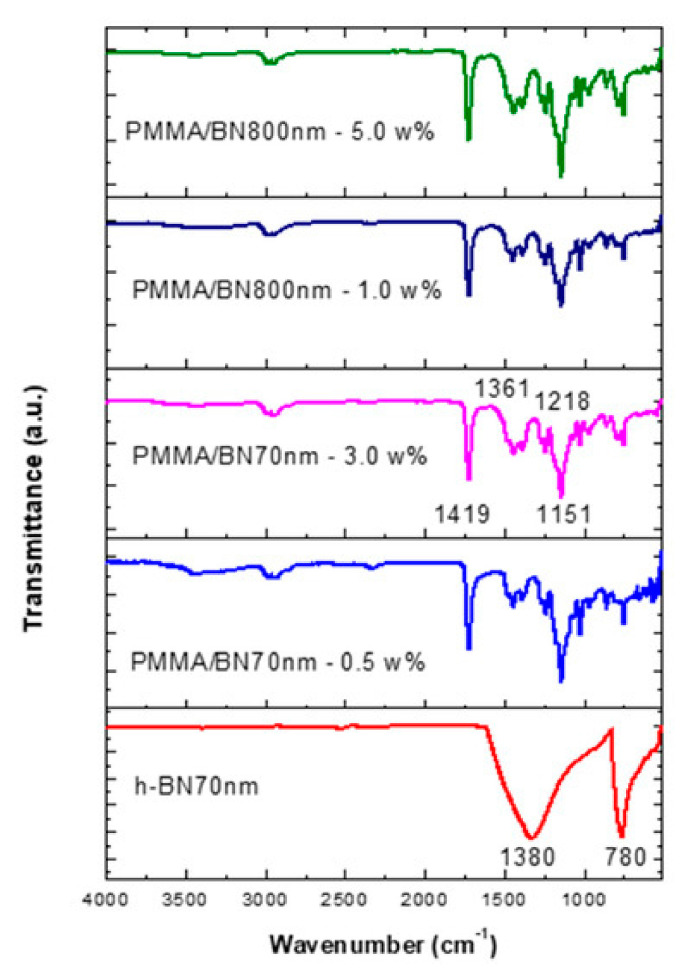
FT-IR data of specimens made by ultrasonic mixing for nano-sized h-BN reinforcement with different concentrations [97].

Figure 2 shows the FT-IR data of samples prepared by mixing UNIFAST III (GC Corporation, Tokyo, Japan) resin powder with ultrasonic-mixed UNIFAST (GC Corporation, Tokyo, Japan) liquid monomer, which includes starting materials and reinforced, nanosized hexagonal boron nitride h-BN (US Research Nanomaterials Co., Ltd., Houston, TX, USA) at various concentrations. Most researchers agree that there are two distinct IR absorption bands in boron nitride films. These are the band around 1380 cm^−1^ (in plane) and the band around 780 cm^−1^ (out of plane), which is due to B–N stretching and B–N–B bending, respectively [97].

## 5. Raman Spectroscopy

### 5.1. Principle of Technique

Raman spectroscopy is a technique used to study vibrational-rotational spectra of molecules. In the light scattered by the examined medium, apart from the component with the same frequency as the incident light (Rayleigh scattering), there are components with a changed frequency. The Raman spectrum is represented as a function of the intensity of the scattered light versus the frequency, (wavenumber), which is calculated as a difference between the frequencies of the incident and scattered radiation (Raman shift).

Two main techniques are used to obtain the Raman spectrum: dispersion and Fourier transform (FT). They differ in the wavelength of the incident radiation and the method of detection. Typically, lasers of different wavelengths result in different penetration depths in the sample surface. The shorter the laser wavelength, the information closer to the surface it provides. On the other hand, when the selected laser wavelength is too long, information about the material near the surface may remain hidden (or omitted). Therefore, the selection of the correct laser wavelength is very important to obtain an accurate Raman characterization result, especially in the case of multi-layer substrates or surface sensitive samples. Currently, many new modifications are used in Raman spectroscopy, which primarily increase the sensitivity of the technique, such as: Resonance Raman Spectroscopy (RRS), Surface Enhanced Raman Spectroscopy (SERS), Confocal Raman Microscopy and Coherent Antistokes Raman Spectroscopy (CARS). Raman spectroscopy provides a lot of important information about the geometric structure of molecules and the nature of chemical bonds. In the case of crystalline samples, among others, information on the structure, crystal lattice arrangement, elastic properties, stresses and the nature of phase transitions can be obtained. Hence, Raman spectroscopy can be used in qualitative analysis, because each compound is associated with a characteristic Raman spectrum, which is a “dactyloscopic trace”, as well as it provides quantitative information based on the dependence of the intensity of signals on the concentration of substances in the analysed sample [56,98,99,100,101].

### 5.2. Type of Tested Samples

In the abovementioned techniques, samples can be tested in a wide variety of states; for example, in the form of solids, liquids or vapours, hot or cold, in bulk, as microscopic particles, or as surface layers. Typical accessories for Raman spectroscopy are powder sample holders, cuvette holders, small liquid sample holders (nuclear magnetic resonance sample tubes), and irregularly shaped object clamps [56].

### 5.3. Sample Characterisctics

In the Raman spectrum, the intensity of the radiation coming from the sample is a linear function of the molar concentration and the thickness of the layer. The Raman spectrum is an oscillating-rotational spectrum that carries information, e.g., about the structure of the test substance and its chemical composition. Raman spectroscopy is now an eminent technique for the characterisation of 2D materials and phonon modes in crystals. Properties, such as number of monolayers, inter-layer breathing and shear modes, in-plane anisotropy, doping, disorder, thermal conductivity, strain and phonon modes can be extracted using Raman spectroscopy [102,103].

For example, Raman mappings confirmed that the graphene nanocoating covering the Biomedical-Grade Ti-6Al-4V alloys showed high structural stability and resistance to mechanical stress and chemical degradation, maintaining >99% coverage after corrosion [104]. Raman spectroscopies have been used to study dental materials were mostly find the degree of conversion (DC) of dental composites, adhesives and setting reaction of cements [103].

### 5.4. Advantages and Limitations

Similar to other spectroscopic techniques in Raman spectroscopy the time of measurement is short and sample preparation is simple. Additionally, the technique is not invasive and the obtained results can be easily and rapidly analysed [56].

For all spectrometer systems using visible incident light, the main disadvantage is the phenomenon of fluorescence. This is bigger problem in the visible region than in UV or near-infrared region. Since Raman scattering is a weak effect a strong excitation source is required to provide a high-power density in the sample. Hence, besides the fluorescence caused by the tested sample, any tiny impurities that are fluorescent can give large interfering fluorescence signals. Since fluorescence occurs at energies below the excitation value, it can be quite intense in the energy region covered by Raman Stokes scattering [56].

### 5.5. Applications

Raman spectroscopy was used to determine the rate of polymerization of bioglass ceramic [105], identification and testing of the presence of various compounds in implant materials [106], ceramics [107,108,109], bioglass [110]; analysis of the phase composition of bioglass [111], ceramics [112,113]; and evaluation of the degree of conversion of resins [114] and ceramics [115].

### 5.6. Spectrum Example

**Figure 3:**
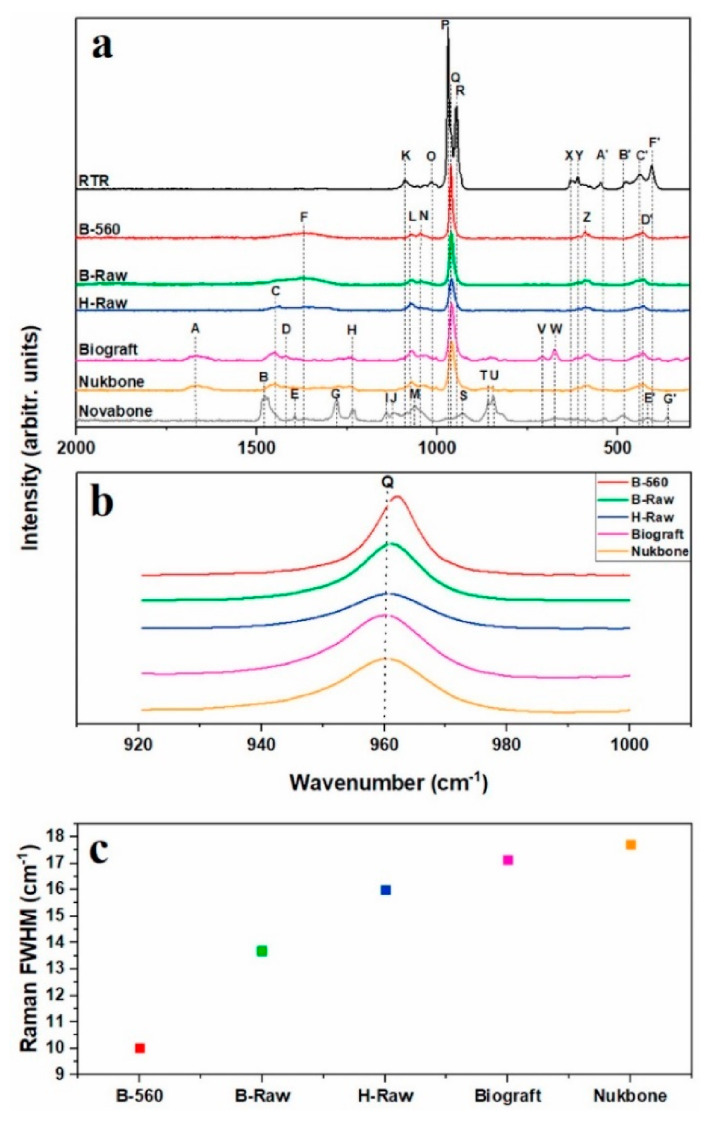
Raman spectra of (**a**) B-560, B-Raw, H-Raw, and commercial bone grafts; (**b**) bands at 960 cm^−1^ of the hydroxyapatites samples, and (**c**) FWHM values of (**b**) bands [106].

Figure 3a shows seven Raman spectra of bovine (B-Raw) and human (H-Raw) bone powders, calcinated bovine bone powder (B-560) and commercial samples based on β-tricalcium phosphate (RTR, Septodont, Saint-Maur-des-Fossés, France), calcium phosphosilicate combined with polyethylene glycol and glycerine (Novabone^®^, Osteogenics Biomedical, Lubbock, TX, USA) and hydroxyapatite (Nukbone^®^, Biocriss, Mexico City, Mexico and Biograft^®^, Biograft, Mexico City, Mexico). All Raman bands are presented in detail in the article [106]. The bands A, H, O, V, and W correspond to the amide, amine, and DNA atomic groups, while the C and F bands are assigned to C–H groups from proteins and fats of the bone. On the other hand, the bands B, E, G, I, S, U, A′, and G′ (groups C–H) in the Novabone are the organic compounds of PEG and glycerol. Bands corresponding to the carbonate group (CO_3_) are designated with D, J, and E′. Inorganic phase bands of the samples are designated with K, L, N, O, P, Q, R, Y, Z, A′, B′, C′, D′, and F′, corresponding to the phosphate group (PO_4_). Finally, the silicate group (SiO_3_) is represented by the bands M and T. Figure 3b refers to the hydroxyapatite band located at 960 cm^−1^. Figure 3c shows the full width at the half-maximum (FWHM) of the bands presented in Figure 3b. It is observed that the crystal size affects the width and size of the peak, which may be an indication of the crystalline quality too [106]. Furthermore, Castillo-Paz et al. [116] observed changes of raw hydroxyapatite obtained from porcine bones during heat treatment with heating rates of 2.5 and 5.0 °C/min between 600 °C and 1100 °C. They found that crystal growth and the transition from nano to a micro-size occurred above 700 °C. The result was a narrowing of the analysed Raman band of the characteristic Raman band at 960 cm^−1^ for ν1 PO_4_^3−^ and a decrease of full width at half the maximum (FWHM), which was confirmed by HR-SEM images and analysis thermogravimetric (TG). A similar effect was observed by Ramirez-Gutierrez et al. [117], who studied the influence of the temperature and sintering time on structural, morphological, thermal, and vibrational properties of hydroxyapatite obtained from pig bone (BHA). They showed that the higher intensity of Raman peaks is associated with an increase in the size of the crystallites. Based on the FWHM, they investigated changes in crystal quality. They found that the FWHM value decreased with increasing crystal quality for samples sintered at 600 °C. Under these conditions, during a longer sintering time, removal of the organic phase takes place without any structural transformation. This treatment preserves the polycrystalline properties of BHA with improved crystal quality. This was confirmed by the results of XRD and SEM.

## 6. Ultraviolet and Visible Spectroscopy (UV-Vis)

### 6.1. Principle of Technique

UV-Vis spectroscopy is one of the oldest instrumental techniques. The analysis is based on the measurement of electron spectra generated during transitions between the energy levels of valence electrons in the spectral range of 200–750 nm. Energy absorption in the UV-Vis range can cause electron transitions from the lowest energy baseline (π and n orbitals) to higher energy levels (anti-bonding π* orbitals). The method provides information on the structure of the studied molecule, the presence of conjugated double bond systems and aromatic systems. The instrument used in UV-Vis spectroscopy is the UV-Vis spectrophotometer [53,58,118].

### 6.2. Type of Tested Samples

Spectroscopic analysis is commonly carried out in solutions, but solids and gases may also be studied. The liquids can be contained in a vessel made of a transparent material such as silica, glass, or plastic, known as a cell or cuvette. Gases can be in similar cells that are sealed or plugged to make them gas-tight. In general, measurements are performed for compounds are demonstrated absorption in the UV-Vis range, which contain chromophores and auxochromes in their structure; within them, there is an electronic transition from the ground to the excited state. Absorption in the UV-Vis range is demonstrated by compounds that contain chromophores and auxochromes in their structure; within them, there is an electronic transition from the ground state to the excited state [53,119,120,121].

### 6.3. Sample Characteristics

According to Beer Lambert’s law, the measurement of the absorbance of a certain substance in a solution is directly proportional to its concentration; hence absorption spectroscopy can be used for quantitative analysis. Therefore, the technique is an excellent analytical tool for the characterization and evaluation of many materials, including biomaterials and dental composites [100].

### 6.4. Advantages and Limitations

UV-Vis spectroscopy is used for both qualitative and quantitative analysis. Its main advantages are its high sensitivity [119,122], high marking precision [119] and selectivity of determinations [58]. The objective measure for the sensitivity of spectrophotometric method is the molar absorptivity ε, corresponding to the λ_max_ of the tested solution. The ε values for the sensitive methods are above 10,000 dm^3^ mol^−1^ cm^−1^, and the ε coefficient with values below 1000 dm^3^ mol^−1^ cm^−1^ corresponds to the less sensitive methods [119,122]. The accuracy of the determination depends on the concentration range and the class of devices used [119]. It is conditioned by the efficiency of absorption and the selectivity of reagents causing a coloured product with the substance to be analysed [58].

Organic compounds with chromophore groups, in which an electronic transition from the ground state to the excited state can occur, are capable of absorbing radiation in the UV-Vis range. Most frequently aromatic systems and groups with multiple bonds (e.g., >C=C<, >C=O, –N=N–, –NO_2_) are chromophores [119].

### 6.5. Applications

UV-Vis spectroscopy has been applied to study fillers for dental adhesive resins [123], to assess the risk of chemical irritation, allergic reactions and oral hypersensitivity due to the elution of polymers [124], to evaluate new co-initiators useful in the radical photopolymerization of dental polymers [94,125]. This technique was also used to characterize the colour and spectral reflectance and thickness oxide layer [126] and resin [127], to evaluate the transparency of ceramics [128,129], and to assesses the degradation rate of CH-based scaffolds which play a key role in endodontic regeneration periodontal regeneration material [130].

### 6.6. Spectrum Example

**Figure 4:**
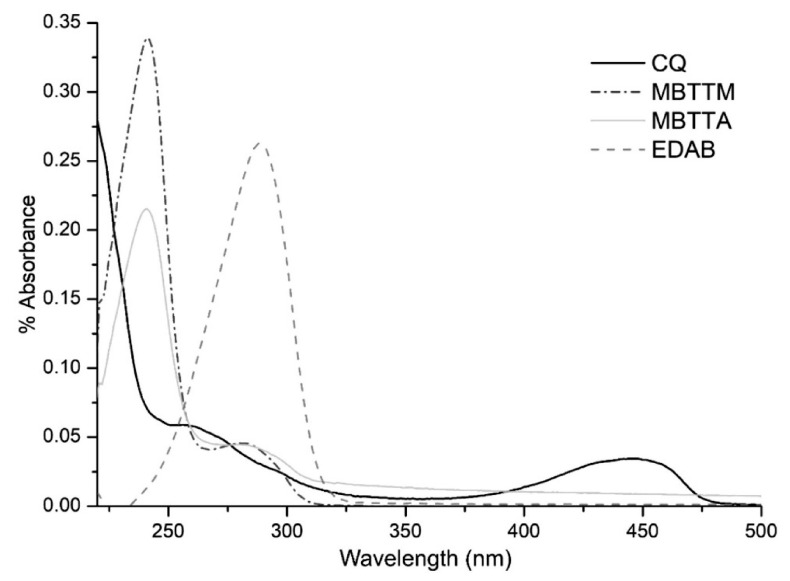
UV-Vis spectra of the CQ initiator and EDAB, MBTTM and MBTTA coinitiators [94].

Figure 4 shows the UV-Vis spectra from the camphorquinone (CQ) and the coinitiators ethyl-4-dimethylaminobenzoate (EDAB), monomers with tertiary amines and four methacrylic (MBTTM) or acrylic (MBTTA) useful in dentistry. The CQ exhibits a strong absorption band in the UV region due to π → π* transition and an absorption band in the visible region at 445 nm (transition n → π*), with a low extinction coefficient. This absorption band at 445 nm gives to CQ the ability to be used as photoinitiator in the visible region, allowing to form an excited state and the subsequent hydrogen abstraction from the coinitiator. Finally, in the case of EDAB, MBTTA and MBTTM used as hydrogen donors, they show only absorption bands in the ultraviolet region, with high extinction coefficient values [94].

## 7. X-ray Spectroscopy

### 7.1. Principle of Technique

X-rays constitute a part of the spectrum of electromagnetic waves covering the wavelength range from 10^−9^–10^−12^ m. They are generated in the X-ray tube during the bombardment of the anode (anti-cathode) with a beam of electrons emitted by the cathode and accelerated in the electric field to a high speed. The X-ray spectrum of the anode material consists of lines associated with the corresponding electron transitions characteristic for a given element. X-ray analysis is possible after splitting it into separate lines. X-rays can be detected with an energy dispersed spectrometer (EDS) or a wavelength dispersed spectrometer (WDS). The analysis can be performed point by point or can be mapped. Elemental analyses are quantified by comparison with standard reference materials.

There are other methods of X-ray spectroscopy that are used in the dental materials testing, such as X-ray absorption spectroscopy (XAS), X-ray emission spectroscopy (XES), X-ray fluorescence spectroscopy (XRF), X-ray Photoelectron Spectroscopy (XPS), and X-ray diffraction (XRD). In the XAS method the relationship between the X-ray intensities before and after passing through the sample of defined thickness is investigated. The XES is the method in which the relaxation products of the interaction of photon or electron beams with the sample is studied. This method is related to the spontaneous emission of X-ray photons in the dipole transition between two electronic states. The XRF is an analytical method that measures the emission of the characteristic secondary or fluorescent X-rays from an excited sample bombarded with a high-energy radiation. Another, relatively new technique is the total-reflection X-ray fluorescence (TXRF), where the total reflection effect is obtained by irradiating the sample, placed on a suitable support (quartz), with the primary radiation from the X-ray tube at an angle smaller than the boundary angle [57,60,131,132,133,134,135].

The XPS method measures the kinetic energy of the emitted electrons from the upper layers of the analysed material (1–10 nm), as a result of the exciting a samples surface with X-rays. XPS allows you to determine what elements are in the ultra-thin layers and thin surface layers of the material, the chemical state and the amount of the element detected. The XRD method is based on measuring the intensity and scattering angles of X-rays, leaving the material with the same energy as the incident radiation. There are many databases of XRD spectra, so that there is rapid phase identification for many different crystal samples [136].

### 7.2. Type of Tested Samples

For testing dental materials, XRF and XRD are mainly used X-ray techniques. The XRF method is used to study metals, ceramics and glass, and the test samples can be of almost any shape and in a form of a powder, paste, solid or liquid. The TXRF method enables the analysis of solutions, thin layers, solids, and different surfaces [137]. Mostly solids are used in XRD testing. The preparation should have a flat and smooth surface. The technique of shaping the preparation consists in filling the window in the aluminium holder with a powder sample and gently pressing the glass plate on the preparation surface [138,139].

### 7.3. Sample Characteristics

Methods using X-rays allow for the analysis of the elemental composition in a small area closely adjacent to or being the surface of the test sample. For example, the effects of high temperature are observed on different restorative dental materials by detecting changes in their microstructural and elemental composition and using X-ray spectroscopy to determine the content of trace elements. The ability to distinguish dental materials by elemental analyses has had an important impact on the identification process [140,141].

### 7.4. Advantages and Limitations

The XRF method is used in both qualitative and quantitative analysis. The advantage of this method is the good selectivity and low quantification limit. The XRF method is of particular importance in the case of surface analysis. In the trace analysis 10^−9^ g of the substance can be determined in a sample weighing about 0.1 g. The technique is non-destructive, which is an additional advantage. The TXRF method can determine trace amounts of elements in the range of 10^−7^–10^−12^ g in 1 g of a sample; small samples (approx. 5·10^−2^ cm^3^) can be used [57,60,131,132,133,134,135].

In the XRF method, it is necessary to use special crystals instead of diffraction gratings; it is only suitable for the analysis of elements with low excitation potential and the presence of matrix effects [57,60,131,132,133,134,135]. XRD is a technique that characterizes only crystalline materials [136].

### 7.5. Applications

The XRF is used to test the composition of materials, the coating thickness, and to identify elements in dental materials: implant alloy [142] and ceramics [143,144,145,146,147,148,149,150,151,152]. XRD is used to obtain information of crystalline structure of solids such as dental ceramics [143,144,145,146,147,148,149,150].

### 7.6. Spectrum Example

**Figure 5:**
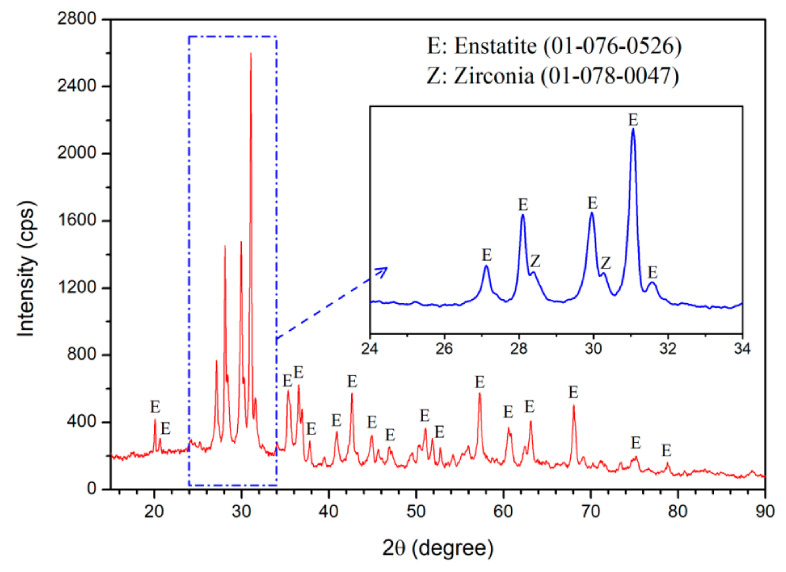
XRD pattern of the glass-ceramic heat-treated at 700 °C for 12 h and then at 1090 °C for 3 min [143].

Figure 5 shows that the crystalline phases resulting from the heat treatment of the glass sample were determined using XRD. Diffraction images were obtained in the range of 2θ from 15° to 90° continuously at 0.6°/min. XRD results proved that spherulite crystals detected in the early phase of crystallization were enstatite [143].

## 8. Mass Spectrometry (MS)

### 8.1. Principle of Operation

Mass spectrometry is an analytical technique used to test or confirm the structure of organic compounds as well as to conduct qualitative and quantitative determination of specific compounds present in a mixture. It enables detecting substances in complex chemical mixtures even in minimal amounts (femtograms). The mode of action of each spectrometer, regardless of its construction, is based on the molecule ionization of the tested substance, allowing for their acceleration in an electric field under vacuum. The heterogeneous flux of positive or negative ions is split into a number of components depending on the mass to charge ratio (*m*/*z*). From the weight to charge ratio of an ion, it is usually possible to deduce the molecular weight of the studied compound or the molecular weight of its fragments. The ionization methods in some mass spectrometers are chosen such that the charge (z) is 1 for most ions, so when interpreting the spectrum, it can be assumed that *m*/*z* corresponds simply to the molecular weight of the ion. Sample ionization in mass spectroscopy can be performed using one of the following methods: Electron Ionisation, (EI) Electrospray (ESI) Fast-Atom Bombardment (FAB), Secondary Ion Mass Spectrometry (SIMS), Laser Desorption (LD), Matrix Assisted Laser Desorption Ionisation (MALDI), and Inductively Coupled Plasma (ICP). Research indicates that secondary ion mass spectrometry (SIMS) is a sensitive technique that characterizes biomaterials and biomineralized bone and dental tissues [153,154,155,156,157].

### 8.2. Type of Tested Samples

Samples such as metals, ceramics, organic and biological materials, polymers, biomaterials, and composites can be analysed. For SIMS testing, samples must be compatible with high vacuum, have a flat surface with minimal topography, and must not contain any “loose parts” that could enter the mass analyser and damage it [156].

### 8.3. Sample Characteristics

Mass spectrometry is an analytical technique based on measuring the mass to electric charge ratio of a given ion. Interpretation of the obtained data allows for the identification of chemical compounds. It enables the determination of their source of origin, precise determination of the composition of complex mixtures of compounds with high molar masses, research on dental materials and polymer chemistry [158,159]. For example, this information can be used to explain the organic composition and eluates of three resin-based pulp coatings in relation to their indications and safety data sheets [159], or to determine the molecular toxicology of substances released by dental materials [160].

### 8.4. Advantages and Limitations

The Time of Flight Secondary Ion Mass Spectrometry (ToF-SIMS) is a qualitative technique used to analyse surface characteristics that provides information related to molecular compounds, typically fragments of much larger organic macromolecules from the outermost surface of the sample [153]. This surface technique is considered highly sensitive.

The SIMS technique combines high mass resolution and the possibility of quantifying elements with the limitation that only a fixed number of predefined ions can be analysed simultaneously (up to seven ions). Due to the high sensitivity of this technique, only high purity solvents should be used [156]. This equipment has limited optical capabilities; experiencing difficulties in collecting positive or negative ion data and, depending on the type of sample, analysis may last from 30 min to 5 h. After testing, the surface of tested sample is left damaged. The technique provides complementary information to XPS technique [153].

### 8.5. Applications

The SIMS technique is used to analyse the composition and chemical interactions of dental resins [161,162], ceramics [161,162,163,164,165] and composite materials [166].

### 8.6. Spectrum Example

**Figure 6:**
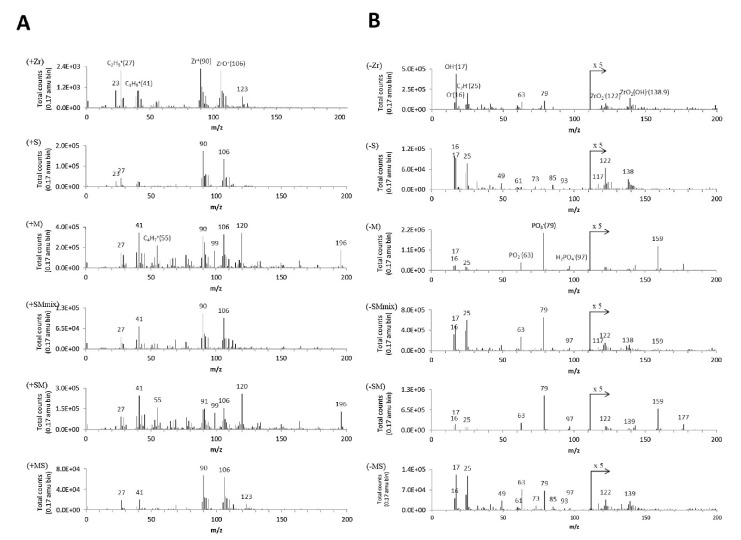
ToF-SIMS spectra (*m*/*z* = 0–200 amu) for the experimental groups. (**A**) The positive ion spectra. The characteristic ion peaks of zirconia are Zr^+^ (*m*/*z* 90), ZrO^+^ (*m*/*z* 106). The ion peaks under *m*/*z* 55 are mainly from organic components. Peaks at *m*/*z* 99 and 120 could be the fragments of MDP monomer. (**B**) In the negative ion spectra, the signals after *m*/*z* 111 were amplified by 5× to reveal the characteristic negative ion peaks of ZrO_2_^−^ and ZrO_2_(OH)^−^ at *m*/*z* 121.9 and 138.9. The characteristic ion SiO_3_C_3_H_9_^−^ (121) in silane is overlapped with ZrO_2_^−^ and thus the peak at *m*/*z* 122 increases [161].

Appropriately prepared zirconium oxide samples together with silane-containing primer (MPS), and 10-Methacryloyloxydecyl dihydrogen phosphate primer (MDP) were subjected to the ToF-SIMS test. Figure 6 shows ToF-SIMS spectra of the studied system. The peaks of Zr^+^ and ZrO^+^ were used as references peaks in the cumulative positive ion spectra (Figure 6A). All groups showed the presence of C_2_H_3_^+^ and C_3_H_5_^+^ derived from organic contamination. All groups show the characteristic ions ZrO_2_^−^ (121.9) and ZrO^−^ (138.9), while many differences are revealed among the five treatments in the negative ion spectra from *m*/*z* of 0 to 200 (Figure 6B). OH^−^ and C_2_H^−^ appear in all spectra, which might be caused by the water adsorption and organic contamination after exposure on air. The increased intensity at *m*/*z* 122 was caused by the accretion of SiO_3_C_3_H_9_^−^ (121) on ZrO_2_^−^ (121.9) [161].

## 9. Summary and Future Aspect

The article presents a review of spectroscopic methods for characterizing surfaces and application in the study of various state-of-the-art dental materials, i.e., ceramics, steel, glasses, cements, composites and resins. The review describes application of different type of spectroscopy such as infrared (FT-IR, and Raman), ultraviolet and visible: (UV-Vis), X-ray (XAS, XES, XRF, XRD) and mass spectrometry (ToF-SIMS).

Table 3 shows the use of spectroscopic methods for studying dental materials, together with the depth of the analysed layer, the nature of information provided (element, depth profile, surface mapping, chemical groups and bonds, etc.) and sample damage (or lack thereof). According to the last criterion, most of the techniques are non-destructive, and some of them—using X-rays for excitation—are almost non-invasive.

As expected, it turns out that not all methods can be generally applied to different types of biomaterial; only some techniques proved to be excellent in certain types of applications. Each method has its own specificity; therefore, the optimization of the approach depends on the correct selection of the method. This is possible due to the wide range of possibilities for characterizing studied materials.

The purpose of this overview is to facilitate the application of these techniques in the field of dental materials orientation in the drawn image. Of course, a single method cannot provide all of required information; in terms of dental materials, no method can provide complete sample information at all levels. Usually, several complementary methods of mutual confirmation and support were used in the study of a given material. However, the complementarity of the provided information were at different levels of elemental composition, chemistry and structure. The research in the field of dental materials has fully benefited from the multi-method approach, which is particularly suitable as no suitable reference materials are yet available for most of the cases. Therefore, the problem of standards has been carefully considered as it provides the most accurate solution to the correction of matrix effects. The latter are inherent especially in the case of thicker preparations, which is usually the most convenient form of preparation of dental materials. We hope this review helps dentists choose the appropriate method and sample preparation, and help the chemist to optimize the experiment setup according to the specimen characteristics.

**Figure 7:**
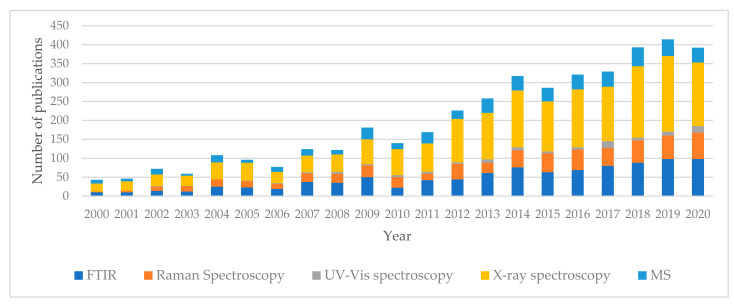
Histogram of the cited publications published in the years 2000–2020 according to Scopus on the application of spectroscopic methods in the analysis of surface phenomena in the study of dental materials and related issues.

The current representative literature review is by no means comprehensive (which would probably grow to around a thousand titles). Figure 7 presents the number of publications on the use of spectroscopic methods in the study of dental materials and related topics based on 4173 titles, indicating that this field is progressively growing.

Within the period 2000–2020, an increase in the number of publications can be noticed. The data suggest that the use of spectroscopic methods to characterize surfaces in the study of dental materials has reached maturity; though it is a highly specialized, the filed shows a high and sustained dynamics. X-ray spectroscopy and FT-IR techniques are the most popular among researchers. Due to the characteristics of these methods, it can be expected their will continue in the upcoming years, especially as dental materials—despite enormous advances made in recent decades—still have many imperfections and the number of experimental objects will increase worldwide.

## Figures and Tables

**Table 1:** The inclusion and exclusion criteria for articles.

Inclusion Criteria	Exclusion Criteria
Research on only dental biomaterials used for restorations.	Literature on dental materials and fluids, equipment used as instruments and equipment for a dental office.
Research including ceramics, calcium phosphates, glasses, polymers, adhesives, composites, glass ionomers, silver amalgam, alloys and titanium implants.	All papers in other than the English language, where the full text was not available.
Dental material research published no later than 5 years ago.	Same data that was published at different times.

**Table 2:** Spectroscopic techniques in different ranges of electromagnetic spectrum radiation.

Region of Electromagnetic Spectrum	Wavelength Range λ (m)	Spectroscopic Technique
Microwave	1–10^−3^	Microwave spectroscopy
Infrared	10^−3^–10^−6^	Infrared spectroscopy Raman spectroscopy
Ultraviolet and visible	10^−6^–10^−8^	UV-Visible spectroscopy Atomic absorption spectroscopy Fluorescence spectroscopy Phosphorescence spectroscopy
X-ray	10^−9^–10^−12^	X-ray diffraction X-ray fluorescence X-ray photoelectron spectrometry Mass spectrometry
γ-ray	10^−12^–10^−14^	Mossbauer spectroscopy

**Table 3:** Comparison of spectroscopic methods for examining the surface of dental materials.

Method	Type of Sample	Analytical Depth	Sample Degradation	Type of Information	Application Examples in Dental Biomaterials and Related Research
Fourier Transform Infrared Spectroscopy (FT-IR)	Gas, liquid, solid	The penetration depth is about 0.5–3 µm [72].	Non-destructive [69]	Quantitative analysis of complex mixtures; the investigation of surface and interfacial phenomena [69]	Implant materials (e.g., to characterize the functional groups of the synthesized apatite particles [82], to study the vibrational states of commercial bone grafts, B-Raw, H-Raw, and B-560 to determine the presence of other functional groups in the samples that do not belong to hydroxyapatite [106]); biopolymers (e.g., characterization of the functional groups in samples of peptide modified demineralized dentin matrix [83]); ceramics (e.g., to complement XRD results, and to determine dental zirconia superficial molecular compositions) [84]; to identify functional groups of HAp nanostructures in resin nanocomposites [85]; recording chemical constituents of implant coatings (e.g., metronidazole decorated Ti interfaces [86]; to detect chemical groups of the modified PEEK films with covalently grafted osteogenic growth peptide [87]); to analyse hydroxyapatite particles without or with immobilized dextranase [88]; bioceramics (e.g., to analyse phase stable β-tricalcium phosphate (β-TCP) in powder samples [89], to determine bulk composition of calcium phosphates [165]); dental resins (e.g., to investigate double bond conversion of dental resin matrix [90] and to calculate the degree of double bond conversion and polymerization rate of photopolymerizable co-initiators in dental monomers [94], to analyse microstructural and surface properties of tricalcium silicate-based pulp capping materials [91], to confirm the final structures of the functional nanoparticles (triazole functional silica) as well as nanocomposites incorporating the functional nanoparticles [92], to analyse powders of monomers: TAT, nt-TiO_2_, and nt-TiO_2_:TAT to evaluate a possible chemical interaction between TAT and nt-TiO_2_ [123]; cements (e.g., to identify the degree of conversion of chemically cured resin modified glass-ionomer cements (RMGICs) testing unset liquids and set materials [93], to provide insight of the setting reactions of a hydraulic calcium silicate cement by taking the FTIR spectra of components before and during the setting reaction [95]; bioglass (e.g., to indicated the integration of the *Calcarea phosphorica* with nano-bioglass ceramic particles [96]); self-curing materials e.g., to compare the structure of boron nitride reinforced PMMA for dental restorations after hand and ultrasonic mixing [97]
Raman Spectroscopy	Gas, liquid, solid (in bulk, as microscopic particles, or as surface layers)	The penetration depth is about 0.01–2300 µm [101].	Non-invasive [56]	Qualitative and quantitative: Investigation of rotational and oscillating spectra of molecules; identification of chemicals component [99]	structure assessment of anti-corrosion coatings e.g., to confirm the growth of graphene and its transfer onto Ti-6Al-4V discs [104]; bioglass (e.g., to investigate the mineral and organic composition of dentin surfaces; demineralized dentin and dentin remineralized with bioglass [110], to analyse the modification of the Ti-Zr-45S5 bioglass alloy surface after oxidation [111]); implant materials (e.g., bovine and human bio hydroxyapatites [106]); ceramics (e.g., chemical analysis of the surface by micro-Raman spectroscopy to establish the presence of MDP monomer on the surface of the zirconia after bonding procedures using MDP containing silane or adhesive [107], to determine the resistance of the titanium substrate to oxidation during the firing of subsequent porcelain layers [108], to assess the chemical composition of the fracture surface in the region of the lithium disilicate ceramic, in the ceramic/staining interface and in the staining applied on the ceramic [109], the complementarily (to XRD) use of micro-Raman to characterize the phase composition of different positions at occlusal loaded area of fixed dental prostheses fabricated from three zirconia grades with varying yttria content [112], to determine phase transformation of the surface of monolithic zirconia submitted to different surface treatments [113], to investigate structural aspects of the glass-ceramic i.e., differently formed crystals, the vitreous area around the crystals, the interface between the TZ3Y substrate and the glass-ceramic, as well as the outer surface of the glass-ceramic [164]; dental resin composites and cements e.g., to evaluate degree of conversion and maximum rate of polymerization [105,114,115]; to analyse powders of monomers: TAT, nt-TiO_2_, and nt-TiO_2_:TAT to evaluate a possible chemical interaction between TAT and nt-TiO_2_ [123]
UV-Vis Spectroscopy	Liquid, solid, gas.	The penetration depth is about 0.02–5 µm [167].	Allows sample recovery [168]	Quantitative: Identification of chemical compounds containing chromophores [168]	resins (e.g., to analyse powders of monomers: TAT, nt-TiO_2_, and nt-TiO_2_:TAT to evaluate a possible chemical interaction between TAT and nt-TiO_2_ [123], to collect optical properties data to calculate colour measurements of dental resin composites containing different opacifiers [127], to investigate the optical properties of Ca_10_(PO_4_)_6_(OH)_2_/Li-BioMOFs structures of resin nanocomposites [85]), polymers (e.g., to determine the maximum absorption of conventional polymethyl methacrylate and the absorption of residual conventional polymethyl methacrylate of specimen eluted in the storage liquid [124]), characterization of co-initiators in photopolymerization of polymers [94,125], oxide layers [126]; ceramics e.g., to analyse the translucency of color-gradient multilayered zirconia, whereas quantitative measurements of translucency can be implemented by analysing the definite transmission of light through each specimen [128,129]
X-ray Spectroscopy	Powder, paste, solid or liquid	The penetration depth: of XRD is about 50–200 mm [169], XPS 1-10 nm, and XRF 0.5–3 μm [170].	Non-destructive and non-invasive [34,171]	Quantitative: Analysis of crystal structure and phase composition [60]	XPS: biopolymers (e.g., chemical composition of peptide-modified demineralized dentin matrix [83]); anti-corrosion coatings (e.g., to confirm that the graphene film was free of copper residues after ammonium persulfate etching [104]); to distinguish and identify dental materials e.g., compomer, glass carbomer, ormocer, giomer, zinc reinforced glass ionomer (GI), silver-alloy reinforced GI, zirconia reinforced GI, and conventional GI using X-ray analysis for obtaining elemental compositions before and after the incineration [141]; bioceramics (e.g., to determine the elemental compositions of the outer layers of calcium phosphates [165]); implant material coatings (e.g., to detect the surface chemical constituents and to confirm the presence of osteogenic growth peptide on PEEK surfaces [87]); XRF: implant alloys e.g., to evaluate the fixture and abutment surface of internal hexagonal connection systems [142] and to evaluate chemical composition of dental ceramics [143,144,145,146,147,148,149,150,151,152]; XRD: implant materials (e.g., to characterize the structure of strontium apatite particles [82]), to measure the crystallinity of hydroxyapatite particles [88], to characterize phase stable β-tricalcium phosphate (β-TCP) [89] or to distinguishing products with the same gross chemical composition but different crystal structures (e.g., different crystal structures of calcium phosphate) [165], to obtain information on the degree of crystallinity of the tricalcium silicate-based pulp capping materials [91]; ceramics (e.g., determination of the crystalline phases in dental zirconia [84,144,146,164], to evaluate phase transformations on the outer surface of fixed dental prostheses fabricated from three zirconia grades with varying yttria content [112], to determine the crystalline phases resulting from the heat treatments of the glass sample in development of strong glass-ceramics based on the crystallization of micron-sized enstatite and nano-sized zirconia and Ti-containing crystals by controlled crystallization of a 51SiO_2_–35MgO–6Na_2_O–4ZrO_2_–4TiO_2_ (mol%) glass) [143]; bioceramics (e.g., to confirm the crystalline nature of nano-bioglass ceramic particles doped with *Calcarea phosphorica* [96], to identify the crystalline and amorphous phases of partially crystallized lithium disilicate ceramics in lithium metasilicate phase [109,143,144,145,146,147,148,149,150], to analyse phase composition of the Ti-Zr-45S5 bioglass alloy [111]; to characterise the phases, the crystallography and the examination of the crystallite size of the Ca_10_(PO_4_)_6_(OH)_2_/Li-BioMOFs [85]; bone grafts (e.g., to identify the crystalline phases and changes in full width at the half maximum (FWHM) of commercial bone grafts, bovine and human bones as well as their BIO-HAps obtained by calcination [106]); self-curing materials (e.g., to observe patterns of boron nitride reinforced PMMA for dental restorations after hand and ultrasonic mixing [97])
Mass Spectrometry	solid	Surface nano-layer [34].	Non-destructive [34]	Qualitative: Composition analysis of solid surfaces and thin films [34]	to precisely determine the composition of complex mixtures of compounds e.g., to elucidate the organic composition and eluates of three resin-based pulp-capping materials [159]; resins [161,162]; ceramics e.g., to analyse the compositions and chemical interactions of the 3-methacryloyloxypropyltrimethoxysilane (MPS)- and 10-methacryloyloxydecyl-dihydrogen-phosphate (MDP)-base primers, in their single or sequential applications, to zirconia [161,162], chemical analysis of saliva contaminated glass ceramic surface and after different cleaning regimens [163], to investigate ion diffusion between the veneer ceramic and the ZrO_2_^−^ based substrate [164], to analyse chemical composition of calcium phosphates [165], composite materials e.g., the release of BPA from two conventional Bis-GMA-containing and two “BPA-free” restorative resin-based composites, which are commonly used as tooth-coloured filling materials, was examined using liquid chromatography—tandem mass spectrometry [166]

## Data Availability

Data sharing is not applicable to this article.
